# Room-Temperature and High-Temperature Tensile Mechanical Properties of TA15 Titanium Alloy and TiB Whisker-Reinforced TA15 Matrix Composites Fabricated by Vacuum Hot-Pressing Sintering

**DOI:** 10.3390/ma10040424

**Published:** 2017-04-18

**Authors:** Yangju Feng, Wencong Zhang, Li Zeng, Guorong Cui, Wenzhen Chen

**Affiliations:** School of Materials Science and Engineering, Harbin Institute of Technology at Weihai, Weihai 264209, China; 13B909079@hit.edu.cn (Y.F.); 16S109250@stu.hit.edu.cn (L.Z.); cuiguorong2010@126.com (G.C.)

**Keywords:** TiBw/TA15 composites, vacuum hot-pressing sintering, microstructure, room-temperature tensile properties, high-temperature tensile properties

## Abstract

In this paper, the microstructure, the room-temperature and high-temperature tensile mechanical properties of monolithic TA15 alloy and TiB whisker-reinforced TA15 titanium matrix composites (TiBw/TA15) fabricated by vacuum hot-pressing sintering were investigated. The microstructure results showed that there were no obvious differences in the microstructure between monolithic TA15 alloy and TiBw/TA15 composites, except whether or not the grain boundaries contained TiBw. After sintering, the matrix microstructure presented a typical Widmanstätten structure and the size of primary *β* grain was consistent with the size of spherical TA15 titanium metallic powders. This result demonstrated that TiBw was not the only factor limiting grain coarsening of the primary *β* grain. Moreover, the grain coarsening of *α* colonies was obvious, and high-angle grain boundaries (HAGBs) were distributed within the primary *β* grain. In addition, TiBw played an important role in the microstructure evolution. In the composites, TiBw were randomly distributed in the matrix and surrounded by a large number of low-angle grain boundaries (LAGBs). Globularization of *α* phase occurred prior, near the TiBw region, because TiBw provided the nucleation site for the equiaxed *α* phase. The room-temperature and high-temperature tensile results showed that TiBw distributed at the primary *β* grain boundaries can strengthen the grain boundary, but reduce the connectivity of the matrix. Therefore, compared to the monolithic TA15 alloy fabricated by the same process, the tensile strength of the composites increased, and the tensile elongation decreased. Moreover, with the addition of TiBw, the fracture mechanism was changed to a mixture of brittle fracture and ductile failure (composites) from ductile failure (monolithic TA15 alloy). The fracture surfaces of TiBw/TA15 composites were the grain boundaries of the primary *β* grain where the majority of TiB whiskers distributed, i.e., the surfaces of the spherical TA15 titanium metallic powders.

## 1. Introduction

A significant amount of attention has been paid to titanium matrix composites (TMCs) due to their superior properties, such as high specific strength, high specific modulus, and high-temperature durability [1,2,3,4,5,6]. In the early stage of studying TMCs, the main preparation method was to add the reinforcing phase directly into the titanium matrix alloy [7]. Due to its own shortcomings (such as the poor bonding between the matrix and the reinforcement, interface reaction, etc.), the ex situ method has not been currently adopted. Therefore, the TMCs fabricated by the in situ synthesis method, in other words, the strengthening phase is formed through the reaction between the elements in the raw materials, which gradually attracted people’s attention [1,2,3,4,5,6,7,8,9,10,11,12,13,14,15,16,17,18,19,20]. The composites prepared by this method have the characteristics of favorable mechanical properties, clean interface, good compatibility and excellent thermodynamic stability. In particular, discontinuously reinforced titanium matrix composites (DRTMCs) fabricated by in situ synthesis methods are sought after due to their excellent hot processing properties, isotropic properties, and low cost [8,9,10,11,12]. The in situ synthesized TiBw reinforcement is recognized as the most compatible and effective reinforcement for titanium monolithic alloy [1,3,13,14,15,16,17]. Among the powder metallurgy (PM) methods, vacuum hot-pressing sintering has been considered as an effective method for preparing in situ synthesis TMCs due to its near-net shape forming, ability for microstructure control, and low material waste [4,13,14,17,18,19]. The previous studies showed that the effects of different types of titanium matrix on TMCs’ performance were very significant. Huang et al. [14] investigated the TiB whisker-reinforced Ti60 titanium matrix composites, and the results showed that composites exhibited better high-temperature performance than that of monolithic alloy and, compared to (TiCp + TiBw)/Ti6242 composites [20], TiBw/Ti60 composites exhibited an obvious improvement in high-temperature tensile strength. TA15 (nominal composition Ti-6.5Al-2Zr-1Mo-1V, wt %) as a near-α alloy of high aluminum equivalent, with excellent process performance, moderate room-temperature and high-temperature strength, good heat stability and welding performance, has a wide application prospects in the aerospace field [21,22,23]. Preparation of TiBw/TA15 composites can maintain the advantages of TA15 alloy and, at the same time, enhance its high-temperature performance. However, there are few published studies on TA15-based composites, and in these few studies only TiC particles are used as the reinforcement [24,25,26]. It is unfortunate that, to the best of author’s knowledge, there is no research on whisker-reinforced TA15 titanium matrix composites. Therefore, it is necessary to study the microstructure and mechanical properties of the whisker-enhanced TA15 titanium matrix composites.

In this work, 0 vol % (monolithic TA15 alloy), 2.5 vol %, 5 vol % TiBw/TA15 composites were fabricated by the in situ synthesis vacuum hot-pressing sintering process, and the main purpose of this paper is to evaluate the influence of TiBw on microstructure evolution, and the tensile properties at room and high temperatures.

## 2. Experimental Procedures

**Figure 1:**
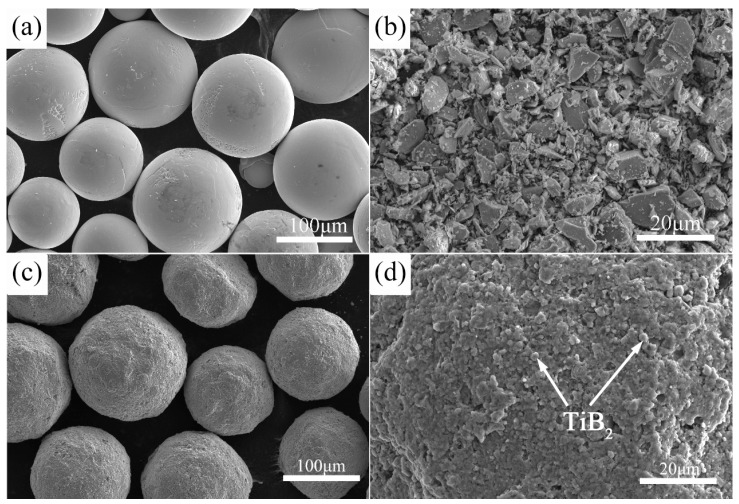
SEM micrographs of raw materials: (**a**) TA15 powders; (**b**) TiB_2_ powders, lower-energy milled powders with low magnification (**c**); and high magnification (**d**); (**c**,**d**) show the mixed powders for fabricating 2.5 vol % TiBw/TA15 composite.

Large spherical TA15 powders and fine prismatic TiB_2_ powders were chosen as raw materials to fabricate the in-situ TiBw/TA15 composites. The size of most spherical TA15 powders is between 75 μm and 250 μm (with an average size of 140 μm) and the size of TiB_2_ powders is between 1 μm and 10 μm, respectively. The TA15 powders, whose chemical composition and particular size distribution are shown in Table 1 and Table 2, were supplied by Shanxi Yuguang Phelly Metal Materials Co. Ltd., Xi’an, China. The TiB_2_ powders, with purity higher than 98%, whose particular size distribution is shown in Table 3, were produced by Zibo Special Ceramics Ltd., Zibo, China. Both powders were mixed by low-energy milling (LEM) in a planetary ball mill (QM-2SP12) at a speed of 100 rpm for a period of 6 h using argon as a protective atmosphere and a weight ratio of balls to powders of 5:1. The morphology of TA15, TiB_2_, and the milled powders are shown in Figure 1a–c, respectively. The aim of the LEM process was not to break down the large TA15 powders but to make the fine TiB_2_ powder adhere to the surface of the large TA15 particles, as shown in Figure 1d. Then the milled powders were vacuum hot-pressing sintered in vacuum (6 × 10^−2^ Pa) at a pressure of 25 MPa, at 1200 °C, for 45 min. After heat and pressure preservation, the composites were cooled at a slow furnace-cooling rate. 0 vol % (Monolithic TA15 alloy), 2.5 vol %, and 5 vol % TiBw/TA15 composites were fabricated by the same process and all samples were 50.5 mm in diameter and 45 mm in height.

**Figure 2:**
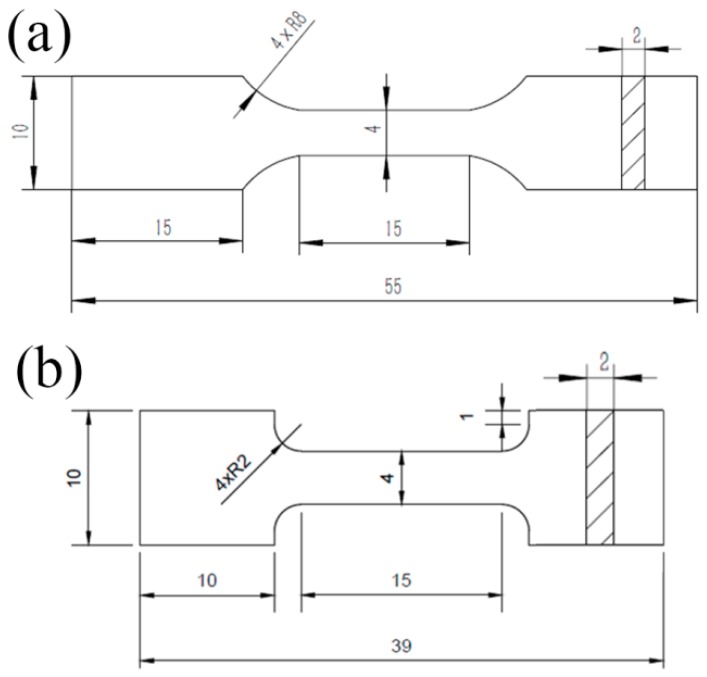
Dimensioned schematic of the room-temperature (**a**) and high-temperature (**b**) tensile specimens (unit: mm).

Phase identification was conducted by X-ray diffraction (XRD) using an XD-2700 unit. Microstructure observation was conducted using scanning electron microscopy (SEM) (Zeiss-MERLIN, Zürich, Switzerland) equipped with an electron back-scattered diffraction (EBSD) detector and TSL OIM 7 analysis software. Before observation, metallographic sandpaper was first used to remove the mark of wire electrodes from the specimen, which was then polished and etched in Kroll's solution (5 vol % HF + 10 vol % HNO_3_ + 85 vol % H_2_O) for 8–10 s. Tensile specimens were cut from the as-sintered samples by electric spark cutting, the gauge sections of the tensile specimens were 15 mm × 4 mm × 2 mm (as shown in Figure 2), and a total of five samples were tested for each sample. Room-temperature tensile tests, which refer to the metal materials testing standards of ISO 6892:1998 [27], were performed on an Instron-5569 universal testing machine (Instron, Norwood, MA, USA), and high–temperature tensile tests, which refer to the metal materials testing standards of ISO 783:1999 [28], were performed on an Instron-1186 universal testing machine at 400 °C, 500 °C, 600 °C, and 700 °C at the same constant crosshead speed of 0.5 mm/min (with a corresponding strain rate of 5.5 × 10^−4^ s^−1^). Hardness was tested under conditions of 1 kg load for 10 s.

## 3. Results and Discussions.

**Figure 3:**
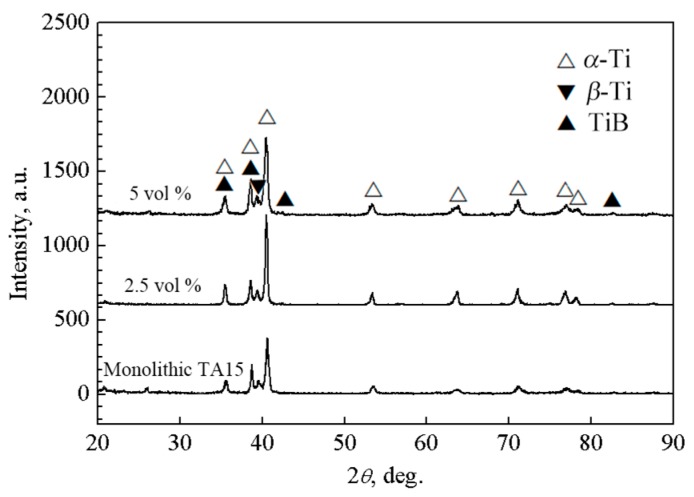
X-ray diffraction patterns of monolithic TA15 alloy and TiBw/TA15 composites with different volume fractions of reinforcement.

Figure 3 shows the XRD patterns of the as-sintered monolithic TA15 alloy, 2.5 vol % and 5 vol % TiBw/TA15 composites. Three types of phases, *α*-Ti, *β*-Ti, and TiB, have been found, and no raw material TiB_2_ was detected. The result demonstrates that TiB whisker-reinforced TMCs can be fabricated by the in situ reaction between Ti and TiB_2_, and similar results have been reported by Huang et al. for TiBw/TC4 and TiBw/Ti60 composites [14,29].

(1) $$Ti+TiB_{2}\to 2TiB$$

**Figure 4:**
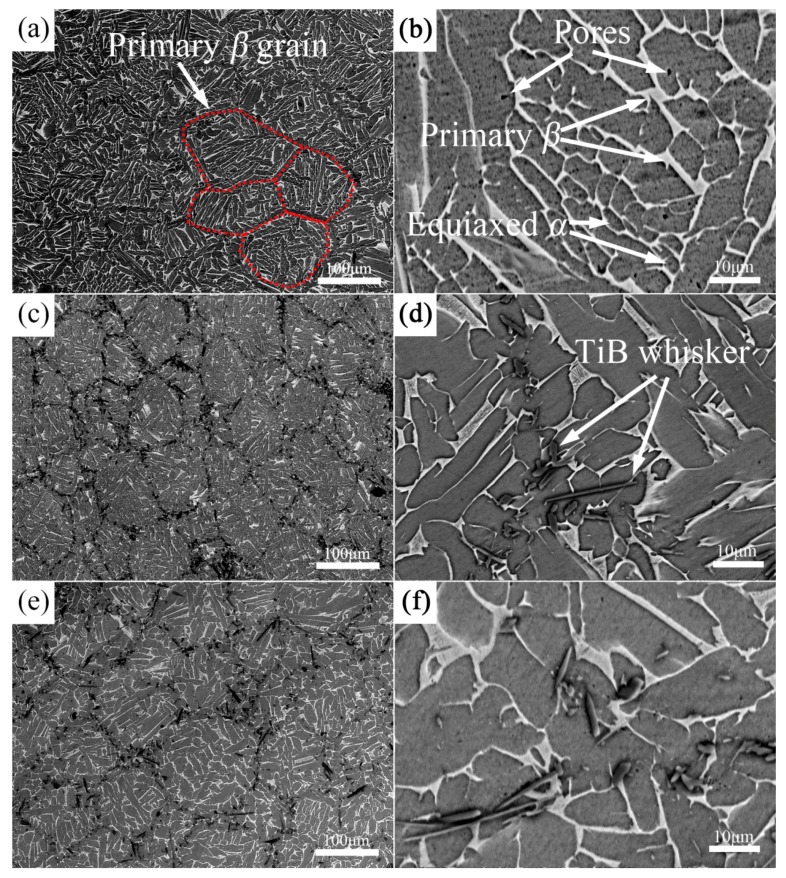
SEM micrographs of 0 vol % (**a**,**b**); 2.5 vol % (**c**,**d**); and 5 vol % TiBw/TA15 (**e**,**f**) with low magnification (**a**,**c**,**e**) and high magnification (**b**,**d**,**f**).

Figure 4 shows SEM micrographs of monolithic TA15 alloy and TiBw/TA15 composites with different volume fractions. All samples present the almost dense structure with low levels of porosity (less than 0.5%, measured in metallographic photos). It can be concluded that 1200 °C, at 25 MPa, are the reasonable sintering parameters in order to fabricate TiBw/TA15 composites with different volume fractions by vacuum hot-pressing sintering.

Figure 4a,b shows SEM micrographs of as-sintered monolithic TA15 alloy at different magnification. It can be seen that the size of the primary *β* grain is consistent with the size of the spherical TA15 titanium metallic powders. This demonstrates that, in the hot-pressing process, the interface of raw material spherical TA15 powders or the impurities on the surface of spherical TA15 powders will limit the growth of the primary *β* grain or else the size of the primary *β* grain will grow larger than the size of spherical TA15 titanium metallic powders. The matrix microstructure presents a typical Widmanstätten structure, the hexagonal close-packed (hcp) *α* phase is dark, and the body-centered cubic (bcc) *β* phase is the lighter constituent. When the TA15 alloy is slowly cooled form a temperature above *β* transus, *α* precipitates would first nucleate and grow along the primary *β* grain boundaries. Upon the further cooling, parallel intergranular *α* plates nucleate from the *β* grain boundary, and grow into lamellas, forming “the typical Widmanstätten microstructure” [30,31], i.e., the coarse grain boundary of the primary *β* grain is clear and complete, presenting as a continuous grain boundary *α* phase, whereas within the primary *β* grain there are transformed *β* phases, presenting as a coarse *α + β* lamellar microstructure. The residual intergranular *β* phase presents a layer morphology interlacing with the primary *α* phase like a basketweave, and the volume fraction is measured to be ~19.6% ± 2.0%. Moreover, there is a small amount of equiaxed *α* phase. This is because the *α* phase exhibits equiaxed or near-equiaxed morphology in the composite when cooled from above *β*-transus temperature to ambient temperature at a low cooling rate [25], and the existence of equiaxed *α* is advantageous for the plasticity of composites [21,32].

With the addition of whiskers, there is no significant difference in the matrix structure (Figure 4c–f except whether or not the grain boundaries of primary *β* contain TiBw. This is because, in the process of low-energy mixing, fine TiB_2_ have been adhered to spherical TA15 metallic powders (as seen in Figure 1c,d). In the subsequent sintering process, an in situ reaction occurs between the TiB_2_ and the Ti around the TiB_2_, forming the TiB whiskers distributed on the surface of the spherical TA15 metallic powders or the grain boundary of the primary *β* grain forming the 3D network microstructure. Similar results have been reported in TiBw/TC4 composites by Huang [18]. As can be seen in Figure 4d, the TiB phase presents whisker-like morphology due to its special B27 crystal structure [14]. It can be also found that with the increase of reinforcement volume fraction (Figure 4d,f), the content of TiBw in the primary *β* boundary increases, resulting in the deterioration in the connectivity of the matrix.

**Figure 5:**
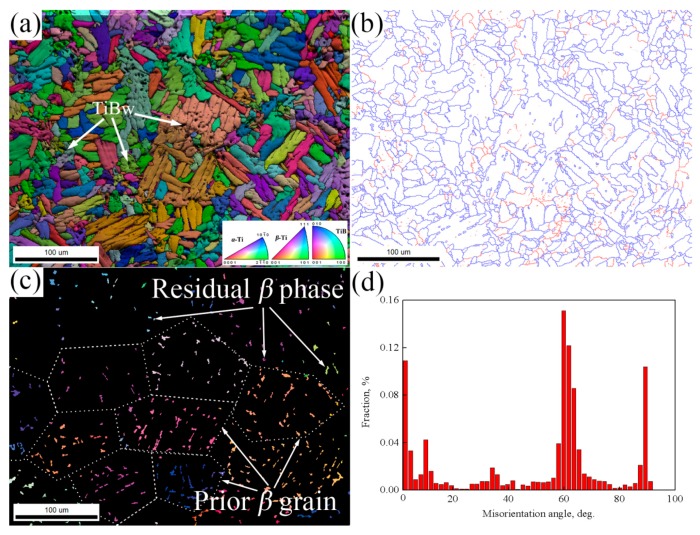
EBSD results of 2.5 vol % TiBw/TA15: (**a**) IPF + IQ + GB; (**b**) EBSD results showing the LAGBs (red lines, 2°–15°) and HAGBs (blue lines, 15°–180°); (**c**) residual *β* phase; (**d**) misorientation angle of *α*-Ti.

As can be seen from the SEM micrographs (Figure 4), there are no obvious differences in the microstructure of the matrix with different reinforcement volume fractions. In order to better understand the microscopic mechanisms of composites fabricated by vacuum hot-pressing sintering, EBSD has been employed to detect the 2.5 vol % TiBw/TA15 composites. Figure 5a shows the IPF + IQ + GB (IPF is the inverse pole figure, IQ is image quality, and GB is grain boundary) of the 2.5 vol % TiBw/TA15 composites, and it can be seen that there are no significant differences compared with the SEM micrographs (Figure 4c,d). The grain coarsening of *α* colonies is obvious, which is due to the slow cooling being beneficial to the growth of *α* colonies [30], and the grain orientation is random, and there is no obvious texture. It is worth noting that there are many fine equiaxed *α* grains distributed at the primary *β* grain boundaries (near the TiBw region). The reason is that the presence of whiskers promotes the growth of equiaxed *α* grains. A similar result has been reported in TiBw/Ti60 composites [33]. The proportion of HAGBs is significantly higher than that of LAGBs, as shown in Figure 5b. Figure 5c shows the IPF of the residual *β* phase, and the residual *β* phase of different primary *β* grains show different colors. The size of the primary *β* grain is consistent with the SEM micrographs (Figure 4c,d). It can be also found that the LAGBs (Figure 5b) are distributed at the grain boundary of the primary *β* grain, and also coincide with the region where whiskers are distributed. This means that LAGBs reflect the angle of *α*-Ti and TiBw. Moreover, there are no LAGBs within the primary *β* grains, indicating that a sufficient recovery is undertaken during the sintering and slow furnace cooling process. Figure 5d shows the misorientation angle of *α*-Ti of 2.5 vol % TiBw/TA15. The peaks of the curve are approximately centered at 10°, 60°, 63°and 90°, illustrating that *α* lamellae inherit the orientation relationship of their parent *β* grain, according to the Burgers relationship. S.C. Wang et al. [34] have reported there are 132 possible types of *α*/*α* boundary and every possible ratio of each type’s reduced angle. According to the data in Figure 5d, the ratio of 10°, 60°, 63°, and 90°, as well as their theoretical calculation ratios, are shown in Table 4, and the actual ratio is close to the theoretical calculation ratio. This result verifies the correctness of the reported literature’s theory.

**Figure 6:**
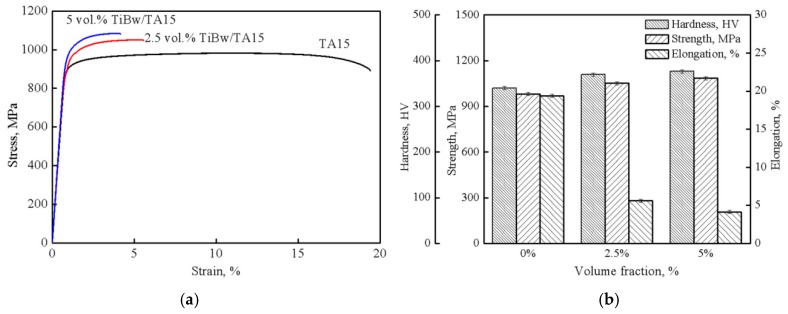
Room-temperature mechanical properties of composites with different volume fractions. (**a**) stress-strain curves of TMCs with different volume fraction of reinforcement; (**b**) mechanical properties of TMCs with different volume fraction of reinforcement.

Figure 6 shows the mechanical properties of composites with different reinforcement volume fractions. When a small amount of whiskers (2.5 vol % TiBw) are added into the TA15 matrix alloy, the strength and the hardness are only slightly increased (7% and 9%), but the plasticity is greatly reduced (only 29% of the TA15 alloy). As the whisker content continues to increase (5 vol % TiBw), the strength and hardness continue to increase, plasticity continues to decrease, but the ranges of change are very small. The reason is that the in situ synthesized TiBw, which are distributed in the primary *β* grain boundaries, can effectively reduce the grain boundary weakening, so the strength and the hardness of the composites will increase [16]. However, with the addition of whiskers, the connectivity of the matrix will deteriorate, resulting in the decrease of the plasticity. Even if the whisker content is 2.5 vol %, the connectivity of the matrix is greatly reduced, as can be seen in Figure 4d. With the increase of TiBw, the content of whiskers distributed in the grain boundaries increases, and the connectivity of the TA15 matrix worsens (as seen in Figure 4c,e), and it is unfavorable for the plasticity, but favorable for the strength and hardness of the composites.

Table 5 shows the room-temperature tensile properties of TA15 composites fabricated by different methods. The tensile properties of 2.5 vol % TiBw/TA15 in this research is better than that of 10 vol % TiC/TA15 fabricated by casting, and there is no significant difference between the 5 vol % TiBw/TA15 fabricated by vacuum hot-pressing sintering and 5 vol % TiCp/TA15 fabricated by lase melting deposited (LMDed), but the composites fabricated by vacuum hot-pressing sintering have a better uniformity than composites fabricated by LMDed. Combined with the above microstructure results (Figure 4), vacuum hot-pressing sintering is an appropriate method for preparing TiBw/TA15 composites.

**Figure 7:**
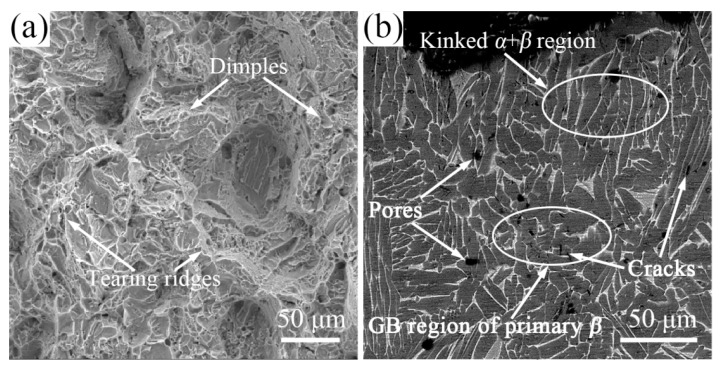

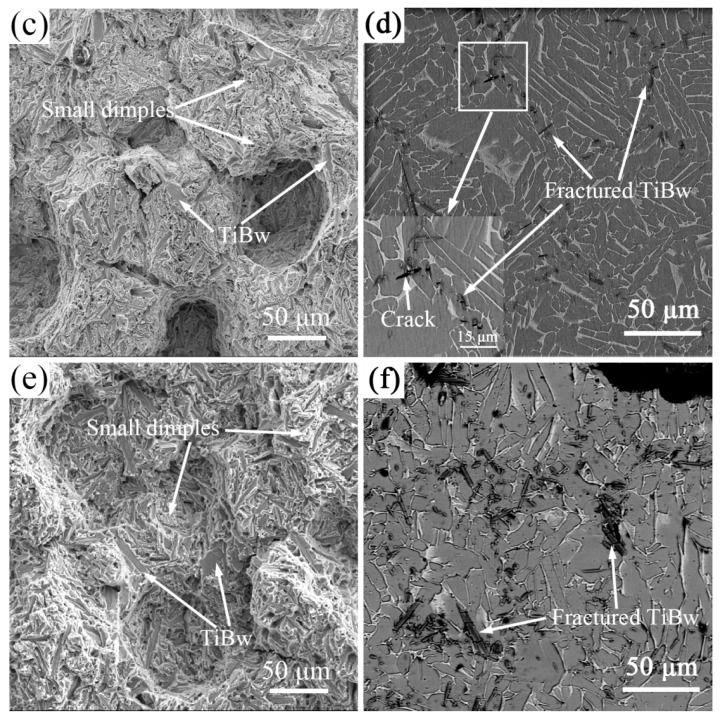
SEM images showing fracture surfaces and longitudinal sections of composites with different volume fractions of reinforcement: 0 vol % (**a**,**b**); 2.5 vol % (**c**,**d**); and 5 vol % (**e**,**f**); and fracture surface (**a**,**c**,**e**) and longitudinal sections (**b**,**d**,**f**).

In order to further investigate the deformation behavior of the matrix and the load-bearing effect of TiBw, as well as the fracture mechanisms at room temperature, fracture surfaces and longitudinal sections near the fracture surfaces of the failed composites with different reinforcement volume fractions are examined, as shown in Figure 7. It can be seen from Figure 7a,b that the monolithic TA15 alloy exhibits ductile fracture characteristics with typical dimple morphologies. The dimples are large and deep, and *α* colonies and the residual *β* layer near the fracture surface become kinked, suggesting a large amount of plastic deformation before rupture and the excellent ductility of the as-sintered monolithic TA15 alloy. It also can be concluded that the coarse *α* colonies have good deformation ability. Therefore, the fracture mechanism of monolithic TA15 alloy is ductile failure. Moreover, there is an interesting phenomenon in Figure 7b that there are cracks and pores in the longitudinal sections near the fracture surfaces of monolithic TA15 alloy. The cracks distribute in the grain boundary of the primary *β* grain. This is the behavior of the crack initiation or propagation during the tensile process. However, the pores distribute within the primary *β* grain. The reason for the existence of pores may be that the pores are the cracks after further deformation. In the initial stage of deformation, the cracks occur in the primary *β* grain boundary and inside the primary *β* grain. When the deformation continues, the cracks at the grain boundaries are merged to form tearing edges, and meanwhile, the cracks inside the grains are blunt by the coarse *α* colonies of the TA15 alloy with good plasticity. This means that crack propagation is restricted and the cracks are pulled along the load direction, forming the pores. The pore is the characteristic of large deformation within the primary *β* grain. It can also be concluded that only the cracks initiating at the primary *β* grain boundary will merge into each other, forming the tearing ridges, indicating that there is a grain boundary weakness in as-sintered TA15 alloy.

When the reinforcement is added (Figure 7c,d), the fracture appears to be an integral spalling and the fracture is concentrated at the interface of the original spherical TA15 powders, and the pores in Figure 7b have disappeared. It can be concluded that the fracture mechanism changes with the addition of TiBw. As can be seen in Figure 7c,d, the whiskers parallel to the tensile load direction are separated from the matrix, and the debonding of the TiBw and matrix is very unfavorable for the ductility of the composites. However, the whiskers perpendicular to the tensile load direction are broken up, indicating that TiBw has good adhesion to the matrix, and these whiskers strengthen the grain boundary of the matrix and contribute greatly to the increased strength of the composites compared to monolithic TA15 alloy (as can be seen in Figure 6). The matrix around the whisker exhibits ductile fracture characteristics with typical dimple morphologies. However, the dimples are smaller and shallower than those of monolithic TA15 (Figure 7a), suggesting low plastic deformations of the matrix before rupture and poor ductility of the composites. The fracture mechanism of the 2.5 vol % TiB/TA15 composites is a mixture of TiBw brittle fracture and matrix ductile failure. It can be also found that there are cracks in the grain boundaries of the primary *β* grain and no pores or cracks within the primary *β* grain. This result indicates small deformations occur during the tensile test, consistent with the SEM image results (Figure 7d) and the tensile test result (Figure 6). From the above results, it can also be inferred that the dislocations migrate along the *α* colonies and will pile up at the interface of the TiB phase and the *α* phase, resulting in stress concentration in this region. With the increase of the tensile degree, the degree of stress concentration increases, causing the debonding of TiBw (parallel to the tensile load direction) and the matrix, and the fracture of TiBw (perpendicular to the tensile load direction, similar to the TiBw of the as-extruded TMCs after tensile testing [16,17]). Since whiskers are concentrated at the grain boundaries, even if the addition of a small amount of whiskers will greatly reduce the matrix connectivity. Moreover, the debonding of TiBw and the matrix is also very unfavorable to plasticity. This is the reason why the elongation is so much reduced, even if very small percentages of amount of TiBw were added (2.5 vol %).

As the volume fraction of the reinforcement increases (Figure 7e,f), the basic features are the same as the 2.5 vol % TiBw/TA15 (Figure 7c,d). It is noteworthy that the content of whiskers distributed at the interface increases, and the dimples are the smallest and shallowest, indicating that there is only a small plastic deformation before fracture. Moreover, the whisker content increases at the interface, resulting in the increase of whiskers parallel to the tensile load direction and perpendicular to the tensile load direction. That is to say, the connectivity of the matrix becomes worse and the number of TiBw which are beneficial for improving strength also increases. Therefore, the plastic reduction is more obvious, and the strength and hardness increase.

**Figure 8:**
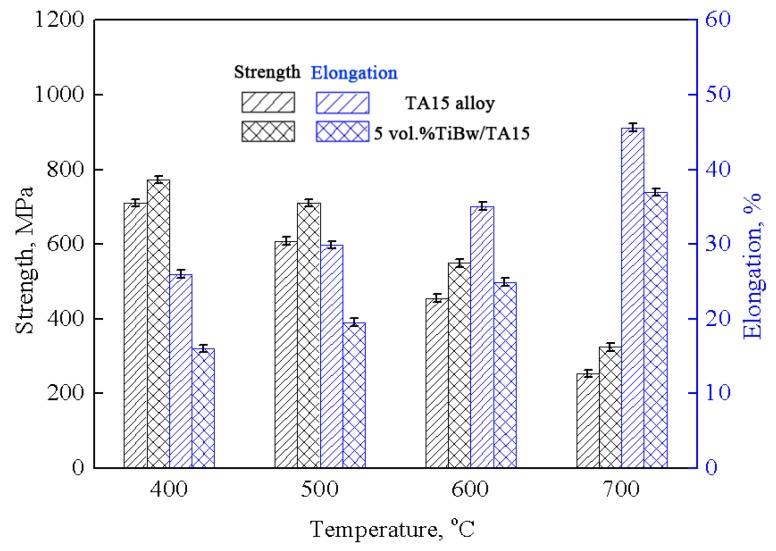
High-temperature tensile properties of the as-sintered monolithic TA15 alloy and 5 vol % TiBw/TA15 composites at 400 °C, 500 °C, 600 °C, and 700 °C.

Figure 8 shows the high-temperature tensile properties of the as-sintered monolithic TA15 alloy and 5 vol % TiBw/TA15 composites at 400 °C, 500 °C, 600 °C, and 700 °C. Due to the limited conditions, the extensometer is not used during the high-temperature tensile process, so there are no yield strengths in the results. Compared to the monolithic TA15 alloy, the tensile strength of 5 vol % TiBw/TA15 composites are increased to 773 MPa, 711 MPa, 549 MPa, and 324 MPa from 710 MPa, 609 MPa, 456 MPa, and 254 MPa at 400 °C, 500 °C, 600 °C, and 700 °C, respectively. That is to say, the tensile strength of 5 vol % TiBw/TA15 can be increased by 10.4%, 16.7%, 20.4%, and 27.5% compared with that of monolithic as-sintered TA15 alloy at 400 °C, 500 °C, 600 °C and 700 °C, respectively. In addition, the tensile elongation of the composites decreases compared to that of the as-sintered monolithic TA15 alloy, and both increase with the increasing drawing temperature. In detail, the tensile elongation of the 5 vol % TiBw/TA15 composites slightly decreases to 16%, 19.5%, 24.9%, and 37% from 26%, 30%, 35.1%, and 45.6% at 400 °C, 500 °C, 600 °C, and 700 °C, respectively. Therefore, the high-temperature tensile properties of composites are enhanced by the addition of the TiBw. The reason for the improvement in the high-temperature tensile strength is that TiBw distributed at the grain boundaries effectively decreases the grain boundary weakening effect at high temperature, and similar results have been reported in TiBw/Ti60 composites [14], TiBw/TC4 composites [35], and TiC/TA15 composites [26]. With the addition of TiBw, the high-temperature tensile elongation of the composites decreases obviously. The reason is the same as for the room-temperature tensile elongation.

Comparing with the tensile properties of 625 MPa, 7.0% and 342 MPa, 18% at 600 °C and 700 °C of 10 vol % TiCp/TA15 composites fabricated by laser melting deposition [26], although the present composites exhibit slightly lower in high-temperature tensile strength, the composites fabricated by vacuum hot-pressing sintering show an obvious improvement in high-temperature elongation. The results indicate that, compared to TiC particles, TiB whiskers can not only can effectively increase the high-temperature strength of TA15 matrix composites, but can also ensure favorable high-temperature deformation capacity.

**Figure 9:**
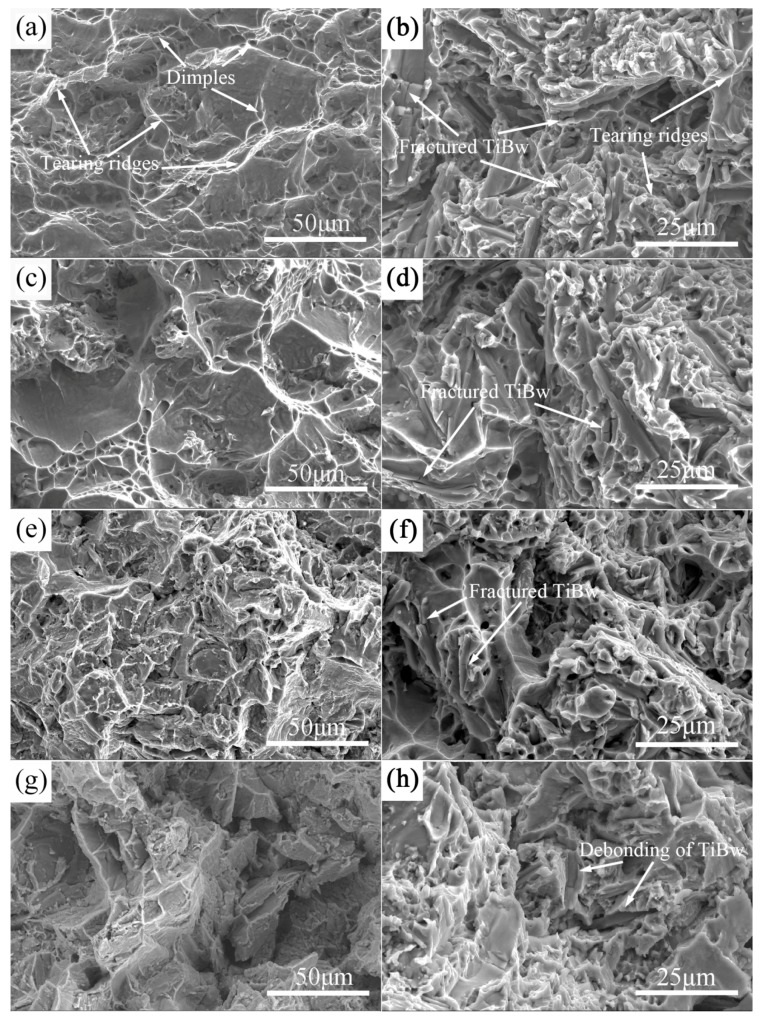
High-temperature SEM fracture of as-sintered monolithic TA15 alloy at 400 °C (**a**); 500 °C (**c**); 600 °C (**e**) and 700 °C (**g**); and of 5 vol % TiBw/TA15 composites at 400 °C (**b**); 500 °C (**d**); 600 °C (**f**); and 700 °C (**h**).

Figure 9 shows the high-temperature fracture surface of as-sintered monolithic TA15 alloy and 5 vol % TiBw/TA15 composites at 400 °C, 500 °C, 600 °C, and 700 °C. For the monolithic TA15 alloy (Figure 9a,c,e,g), as the temperature increases, the depth of dimples and tearing ridges increases, indicating there is a large deformation before fracture, and the fracture mechanism of monolithic TA15 is a ductile fracture.

For the 5 vol % TiBw/TA15 composites (Figure 9b,d,f), the fracture mechanism of the composites was predominantly controlled by whisker fracturing, followed by ductile failure of the matrix in the whole temperature range of 400 °C to 600 °C. Moreover, it can be clearly seen that a large number of TiBw are fractured during the tensile process at high temperature, which indicates the excellent strengthening effect of the TiBw reinforcement due to the strong interfacial bonding of TiBw and the matrix. Additionally, there are large dimples and tearing ridges around the TiBw reinforcement corresponding to the superior tensile elongations of 5 vol % TiBw/TA15 composites at high temperature. However, when the stretching temperature rose to 700 °C (Figure 9h), there were no broken whiskers in the fracture surface, and more TiBw are pulled out from the matrix, i.e., the debonding of the TiBw and the matrix. This is mainly due to the degree of matrix softening increasing at higher temperatures, leading to a reduction of the interfacial bonding strength between the matrix and reinforcement. In general, as the stretching temperature increases, the number and the depth of dimples and tearing ridges increase. The fracture mechanism is a mixture fracture and as the stretching temperature increases, the phenomenon of debonding is more and more due to the softening of the matrix.

## 4. Conclusions

(1) Conditions of 1200 °C and 25 MPa are the reasonable sintering parameters in order to fabricate monolithic TA15 alloy and TA15 matrix composites by vacuum hot-pressing sintering.
(2) There are no significant differences in the microstructure of monolithic TA15 alloy and TiBw/TA15 composites. The matrix exhibits a typical Widmanstätten microstructure, and the size of the primary *β* grain is consistent with the size of spherical TA15 titanium metallic powders. This result shows that, in the as-sintered TA15 alloy (TiBw/TA15 composites) fabricated by vacuum hot-pressing sintering using large-sized spherical TA15 titanium metallic powders (and fine TiB_2_ powders), the grain boundary of the primary *β* grain/interface of the spherical TA15 titanium metallic powders or the impurities on the surface of spherical TA15 powders is the key factor in limiting the grain coarsening of the primary *β* grain.
(3) After sintering, the matrix of the composites consists of not only a coarse *α + β* lamellar microstructure, but also a small amount of equiaxed *α* phase. The equiaxed α distribution coincides with the distribution of TiB whiskers, and both distribute in the primary *β* grain boundary. This result indicates the TiBw are beneficial to the formation of equiaxed *α* grains.
(4) When phase transformation occurs during the cooling process, the β→α is oriented according to the Burgers relationship, the number fraction of the α/α boundary angle inherited from the same parent β grain is consistent with the theoretical calculation.
(5) With increasing volume fractions of TiBw reinforcement, the tensile strength of TiBw/TA15 composites increases and the ductility continues decreasing. The fracture mechanism was changed to a mixture of brittle fracture and ductile failure (composites) from ductile failure (monolithic TA15 alloy).
(6) TiBw can effectively improve the high-temperature strength of the composites. For 5 vol % TiBw/TA15 composites, there is an increasing damage of interface debonding with the stretching temperature. The fracture mechanism of the composites is dominated by TiB whisker fracturing or debonding followed by ductile fracture of the matrix in the whole temperature range of 400 °C to 700 °C. However, there is no fractured TiBw in the composites when the tensile temperature exceeds 700 °C.

## Figures and Tables

**Table 1:** Chemical composition of TA15 powders (wt %).

Al	Mo	V	Zr	Fe	Si	O	C	N	H	Ti
6.62	1.70	2.25	1.9	0.04	0.02	0.15	0.003	0.007	0.001	Bal.

**Table 2:** Size distribution of TA15 powders (diameter).

Size Distribution of TA15 Powders	D10	D50	D90
Diameter (μm)	90	140	200

**Table 3:** Size distribution of TiB_2_ powders (length).

Size Distribution of TiB_2_ Powders (μm)	>8	5~8	2~5	<2
Proportion (%)	11.3	30.9	49.3	8.5

**Table 4:** The ratio of 10°, 60°, 63°, and 90° and their theoretical calculation ratios.

Angle of *α*/*α* Boundary in a Parent *β* grain, ° (Tol ± 1°)	Theoretical Ratio, % [34]	Actual Ratio, %	Actual Misorientation Angle Ratio, %
10 (10.53)	9.1	8	0.04
60 (60,60.83)	54.6	54	0.27
63 (63.26)	18.2	18	0.09
90 (90)	18.2	20	0.10

**Table 5:** Room-temperature tensile properties of the TiB(TiC)/TA15 composites.

Sample	UTS, MPa	Elongation, %
Vacuum hot-pressing sintering 2.5 vol % TiBw/TA15	1051 ± 6	5.6 ± 0.5
Vacuum hot-pressing sintering 5 vol % TiBw/TA15	1085 ± 5	4.2 ± 0.3
Laser melting deposited 5 vol % TiC/TA15 [24]	1086 ± 45	4.3 ± 1.8
As-cast 10 vol % TiC/TA15 [25]	1048 ± 5	3.9 ± 0.6
